# Manifestation of Headache Affecting Quality of Life in Long COVID Patients

**DOI:** 10.3390/jcm12103533

**Published:** 2023-05-18

**Authors:** Kana Fujita, Yuki Otsuka, Naruhiko Sunada, Hiroyuki Honda, Kazuki Tokumasu, Yasuhiro Nakano, Yasue Sakurada, Mikako Obika, Hideharu Hagiya, Fumio Otsuka

**Affiliations:** Department of General Medicine, Graduate School of Medicine, Dentistry and Pharmaceutical Sciences, Okayama University, Okayama 700-8558, Japan

**Keywords:** COVID-19 aftercare clinic (CAC), headache, quality of life (QOL), long COVID, post COVID-19 condition (PCC)

## Abstract

**Objectives**: The present study aimed to elucidate the characteristics of long COVID patients with headaches. **Methods**: A single-center retrospective observational study was performed for long COVID outpatients who visited our hospital from 12 February 2021 to 30 November 2022. **Results**: A total of 482 long COVID patients, after excluding 6, were divided into two groups: the Headache group of patients with complaints of headache (113 patients: 23.4%) and the remaining Headache-free group. Patients in the Headache group were younger (median age: 37 years) than patients in the Headache-free group (42 years), while the ratio of females (56%) in the Headache group was nearly the same as that in the Headache-free group (54%). The proportion of patients in the Headache group who were infected in the Omicron-dominant phase (61%) was larger than the proportions of patients infected in the Delta (24%) and preceding (15%) phases, and that trend was significantly different from the trend in the Headache-free group. The duration before the first visit for long COVID was shorter in the Headache group (71 days) than in the Headache-free group (84 days). The proportions of patients in the Headache group with comorbid symptoms, including general fatigue (76.1%), insomnia (36.3%), dizziness (16.8%), fever (9.7%), and chest pain (5.3%) were larger than the proportions of patients in the Headache-free group, whereas blood biochemical data were not significantly different between the two groups. Interestingly, patients in the Headache group had significant deteriorations of scores indicating depression and scores for quality of life and general fatigue. In multivariate analysis, headache, insomnia, dizziness, lethargy, and numbness were shown to be involved in the quality of life (QOL) of long COVID patients. **Conclusions**: The manifestation of headaches related to long COVID was found to have a significant impact on social and psychological activities. Alleviation of headaches should be a priority for the effective treatment of long COVID.

## 1. Introduction

The number of patients with coronavirus disease 2019 (COVID-19) due to severe acute respiratory syndrome coronavirus 2 (SARS-CoV-2) has been reported to be 762 million worldwide, including 33 million in Japan, as of April 2023 and based on the WHO COVID-19 Dashboard. A few months after the acute symptoms of COVID-19, COVID-19 can subsequently induce prolonged symptoms called long COVID, post-acute sequelae of COVID-19, or post-COVID-19 condition (PCC) [1]. A recent study has shown, by referring to pre-infected and uninfected conditions, that persistent COVID-19 symptoms occurred in 12.7% of infected cases [2].

We have established an outpatient clinic specialized for long COVID in our university hospital in Japan. Patients with long COVID have various symptoms and signs in the post-acute state of COVID-19. Typical symptoms include not only generalized symptoms such as malaise, myalgia, respiratory symptoms, and mental and neurological symptoms but also other specific symptoms, such as taste and smell disorders, hair loss, palpitation, and abdominal discomfort [3,4,5]. Among the various neurological symptoms, including memory impairment, poor concentration, depression, and sleep disorders, a persistent headache is one of the most frequent symptoms in patients with long COVID [6,7,8]. The proportion of patients who experienced persistent headaches beyond the acute phase has been approximately estimated to be as high as 25% [9].

Recently, we revealed that the symptoms of general fatigue, sleep disturbance, and headache increased after the Omicron variant spread worldwide [8]. Another study showed that headache was the second-most frequent symptom in teenagers with long COVID [10]. A persistent headache for more than three months after an infection is considered a diagnosis of “chronic headache attributed to systemic viral infection” [11,12,13]. Since headache is one of the most common symptoms during infection and in long COVID patients [14,15], it is highly possible that headache-related symptoms can cause physical and mental problems in patients with long COVID.

The present study aimed to clarify the characteristics of headaches in patients with long COVID by focusing on the patients’ backgrounds and clinical conditions and to know the impact of headaches on the quality of life (QOL) in long COVID patients.

## 2. Methods

### 2.1. Study Design and Patients’ Characteristics

**Figure 1:**
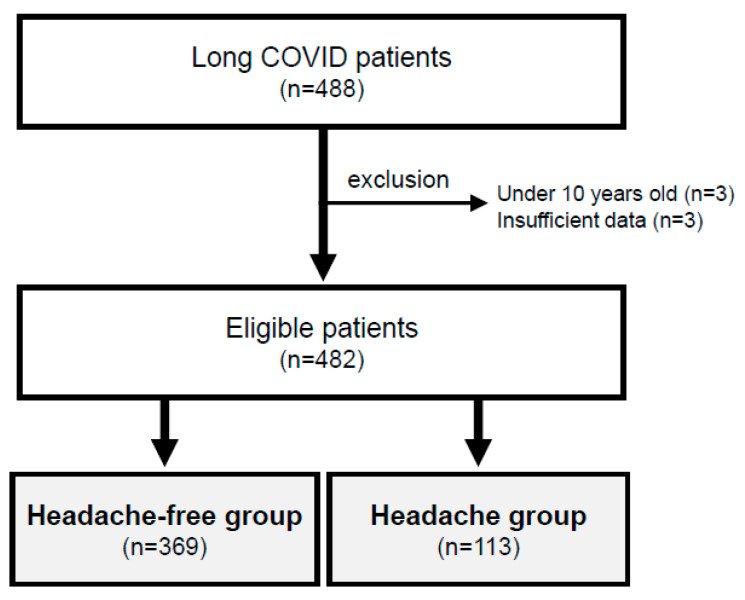
Groups of patients with and without headaches. After excluding 6 of the 488 long COVID patients, 482 patients were compared by dividing them into a Headache group and a Headache-free group.

This study was a retrospective observational study conducted in a single hospital. We enrolled and reviewed the medical records of all long COVID patients who visited the COVID-19 aftercare clinic (CAC) of the Department of General Medicine at Okayama University Hospital in Japan during the period from 12 February 2021 to 30 November 2022 after excluding those whose consent could not be obtained (Figure 1). Those who were under ten years of age and those who had insufficient available data were excluded from the analysis. In the medical practice of the CAC, well-trained generalists evaluate each patient through face-to-face medical interviews and discuss the cases once a week in clinical meetings for the CAC. Although there are various definitions, long COVID was defined in the present study as symptoms that persist for more than four weeks after the onset of COVID-19 [16]. Based on face-to-face medical interviews, patients who complained of headaches at the time of visiting the CAC were defined as a “Headache group”, and those who did not complain of headaches were defined as a “Headache-free group” (Figure 1). We obtained information on age, gender, height, weight, body mass index (BMI), current habits of smoking and drinking, the severity of COVID-19, estimated variants of SARS-CoV-2 as explained below, days from the onset of COVID-19 to the first visit to the CAC, symptoms during the time course and scores of self-rating scales.

The severity of the acute phase of COVID-19 was categorized according to the criteria defined by the Ministry of Health, Labour and Welfare in Japan [17]. Clinical symptoms of long COVID were identified through a physician’s careful medical interview. Based on the epidemiological aspects of COVID-19 in Okayama Prefecture in Japan, the onset of COVID-19 in the patients was divided into three groups: the preceding period, the Delta-dominant period, and the Omicron-dominant period [8].

### 2.2. Blood Chemistry Examination

Selection of the biochemical examination for the long COVID patients was determined by each physician to differentiate various diseases. When laboratory data were required for each medical examination, blood cell counts, inflammatory and coagulation markers, liver and renal functions, electrolytes (sodium, potassium, and calcium), vitamins (vitamin B1, B12, and folic acid), lipids (cholesterols and triglycerides), and hormones (thyroid hormone, adrenocorticotropic hormone, cortisol, growth hormone, and insulin-like growth factor (IGF)-I) were examined, and the obtained data were statistically analyzed. Blood collection was performed in a relaxed sitting position in the late morning to early afternoon when the patients visited our clinic. The auto-analyzer system in the central laboratory of our facility was used to analyze blood samples.

### 2.3. Definition of Self-Rating Scales

Patients were asked to complete questionnaires including a questionnaire for self-rating depression scale (SDS) scoring to assess the respondent’s depressive status [18], frequency scale (FSSG) for the symptoms of gastroesophageal reflux disease (GERD) scoring to assess GERD symptoms [19], the Japanese version of fatigue assessment scale (FAS) scoring to assess fatigue status [20], and the Japanese version of Euro QOL 5-dimensions 5-levels (EQ-5D-5L) scoring to measure health-related quality of life (QOL) [21]. In EQ-5D-5L, a score of “0” indicates the worst QOL, and a score of “1” indicates the best QOL. A score of less than 0.8 points was defined as an impairment of QOL in this study [22].

### 2.4. Statistical Analysis

All statistical analyses were conducted by using EZR, version 1.6 (Saitama Medical Center, Jichi Medical University, Saitama, Japan), a graphical user interface for R, version 4.2.2 (The R Foundation for Statistical Computing, Vienna, Austria) [23]. To compare the clinical backgrounds of patients in the Headache group and the Headache-free group, the Mann–Whitney U test and Pearson’s χ^2^ test were performed for non-normal distributed variables and for categorical variables, respectively. Multiple regression linear analysis was conducted with the EQ-5D-5L score as the outcome variable and each of the symptom variables as the exposure variable. The thresholds for statistical significance were defined as * *p* < 0.05 and ** *p* < 0.01.

### 2.5. Ethical Approval

This study received approval from the Ethics Committee of Okayama University Hospital (No. 2105-030) and followed the Declaration of Helsinki. On the website and the clinic walls in our hospital, we provided information on the protocol of this study. Informed consent from the patients was concluded to be unnecessary due to the anonymization of the patients’ data by the Ethics Committee of Okayama University Hospital, but they were given the option to opt out if they wished. The Strengthening the Reporting of Observational Studies in Epidemiology (STROBE) checklist was completed for this study [24].

## 3. Results

In the present study, six patients with long COVID, including three patients who were under ten years of age and three patients who had insufficient available data, were excluded. The eligible 482 cases were classified into two groups (Figure 1): The Headache group (113 patients: 23.4%) and the Headache-free group (the remaining 369 patients: 76.6%) (Table 1). The median age of the long COVID patients was significantly younger in the Headache group than in the Headache-free group (37 years vs. 42 years). The ratio of females in the Headache group (F/M: 56%/44%) was not significantly different from that in the Headache-free group (54%/46%). Overall, there were no significant differences in other background factors, including height, weight, BMI, gender, smoking habits, and alcohol drinking habits between the two groups (Table 1).

**Figure 2:**
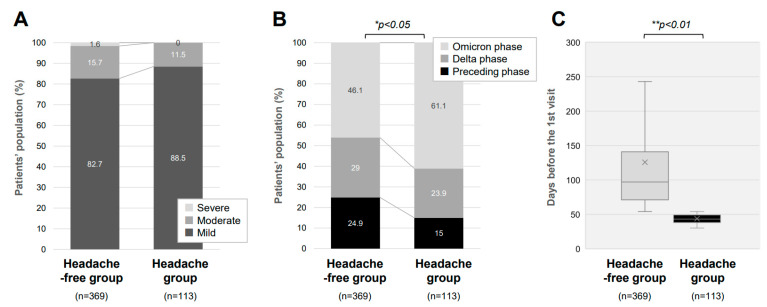
Clinical characteristics of long COVID patients with headache. (**A**) Proportions of patients (%) with different severities of the acute phase of COVID-19 are shown by the presence or absence of long COVID headaches. (**B**) Proportions of patients (%) infected with different variants of SARS-CoV-2 are shown by the presence or absence of long COVID headaches. (**C**) Median durations from the onset of COVID-19 to the first visit to our clinic for patients with and those without long COVID headaches. * *p* < 0.05 and ** *p* < 0.01 indicate statistically significant differences.

As shown in Figure 2A, in the acute phase, most of the long COVID cases had mild severity both in the Headache group (88.5%) and the Headache-free group (82.7%). There was no significant difference in the severity of the acute phase of COVID-19 between the two groups (*p* = 0.199). Of note, it was revealed that the percentage of patients suffering from headaches was significantly higher in patients infected in the Omicron-dominant period than those infected in the other phases of the viral variants (Figure 2B). Namely, patients in the Headache group were predominantly infected in the Omicron-dominant phase (61%) than in the Delta-dominant phase (24%) and the preceding phase (15%) (Figure 2B). This trend significantly differed from that in the Headache-free group, with a smaller percentage of patients infected in the Omicron-dominant phase (46%). Of interest, the time lag before the first visit for long COVID was shorter in the Headache group than in the Headache-free group (Figure 2C). The duration before the first visit to the CAC was significantly shorter in the Headache group (71 days) than in the Headache-free group (84 days).

**Figure 3:**
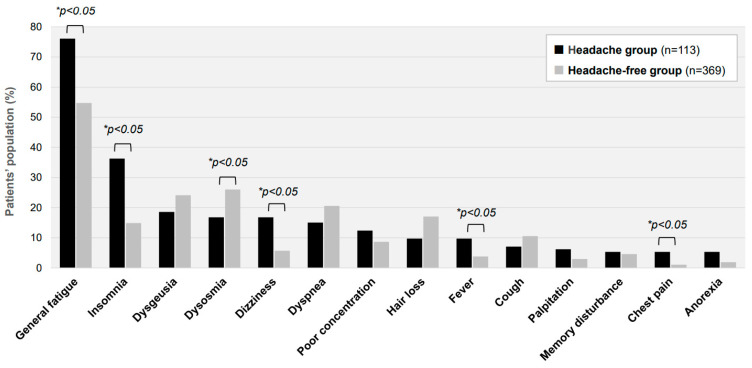
Major symptoms accompanying headache in long COVID patients. The proportions of patients complaining of each long COVID symptom other than a headache in the Headache group (*n* = 369) and the Headache-free group (*n* = 113) are shown. * *p* < 0.05 indicates a statistically significant difference.

As shown in Figure 3, the presence of other comorbidity symptoms accompanying headaches was analyzed in the long COVID patients. The proportions of patients in the Headache group with general fatigue, insomnia, dizziness, fever, and chest pain were 76.1%, 36.2%, 16.8%, 9.7%, and 5.3%, respectively. The proportions of patients in the Headache-free group with those symptoms were 54.7%, 14.9%, 5.7%, 3.8%, and 1.1%, respectively. There were notable differences between the two groups in the proportions of patients with general fatigue and insomnia. In addition, the blood chemistry examinations obtained from 458 of the 482 patients showed that blood cell counts, inflammatory and coagulation markers, lipids, and electrolytes were not significantly different between the two groups. Several biochemical and hormonal markers, including serum levels of alanine aminotransferase (ALT), free thyroxine (FT4), and IGF-I, were found to be slightly higher in the Headache group than in the Headache-free group (21.0 IU/L vs. 17.0 IU/L; 1.33 ng/dL vs. 1.25 ng/dL; and 162.0 ng/mL vs. 144.5 ng/mL, respectively).

**Figure 4:**
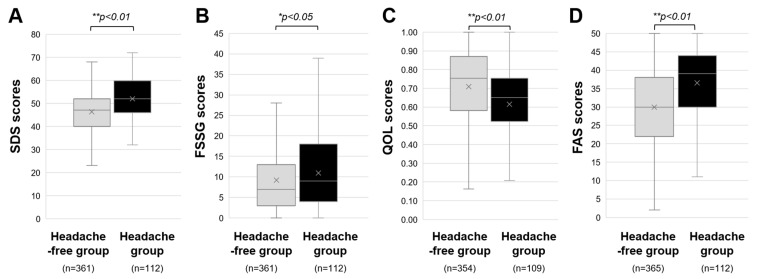
Influence of headache on social and psychological activities of long COVID patients. The medians and interquartile ranges of (**A**) self-rating depression scale (SDS) scores, (**B**) frequency scale for the symptoms of GERD (FSSG) scores, (**C**) Euro QOL 5-dimensions 5-levels scores for measuring quality of life (QOL), and (**D**) fatigue assessment scale (FAS) scores in the Headache group and Headache-free group are shown. * *p* < 0.05 and ** *p* < 0.01 indicate statistically significant differences.

Finally, as shown in Figure 4, patients in the Headache group had significantly higher SDS, FSSG, and FAS scores than those patients in the Headache-free group. The median score of SDS in the Headache group was 46, significantly higher than the median score of 40 in the Headache-free group. The median scores of FSSG and FAS in the Headache group (4 and 30, respectively) were also significantly higher than those in the Headache-free group (3 and 22, respectively). Also, the QOL score was significantly lower in the Headache group (0.54) than in the Headache-free group (0.61).

**Figure 5:**
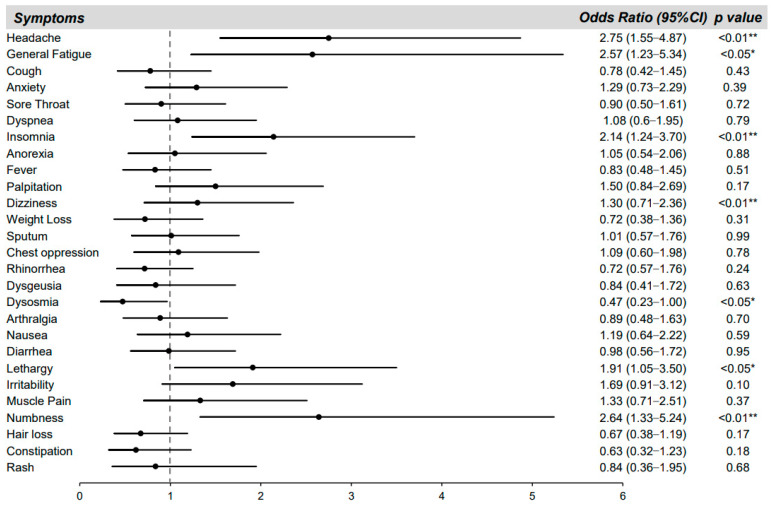
Key symptoms affecting the quality of life (QOL) of long COVID patients. The odds ratios (black circles) and 95% confidence intervals (solid lines) for each symptom during the whole period after COVID-19 for QOL decline (defined as Euro QOL 5-dimensions 5-levels scores < 0.8) are shown. * *p* < 0.05 and ** *p* < 0.01 indicate statistically significant differences.

We further analyzed key symptoms affecting QOL of the long COVID patients (Figure 5). To investigate the actual symptoms affecting the patient’s QOL, all of the manifestations during the infection periods were incorporated into a logistic regression model. Missing values of 9 patients from SDS, 9 patients from FSSG, 19 patients from QOL, and 5 patients from FAS were excluded. In the multivariate analysis, headache, insomnia, dizziness, lethargy, and numbness, but not dysosmia, were significantly related to the QOL of long COVID patients. The odds ratios for the key symptoms affecting QOL of the long COVID patients were 2.75 for headache (*p* < 0.01), 2.57 for general fatigue (*p* < 0.05), 2.14 for insomnia (*p* < 0.01), 1.30 for dizziness (*p* < 0.01), 1.91 for lethargy (*p* < 0.05), and 2.64 for numbness (*p* < 0.01). The odds ratio of dysosmia was 0.47 (*p* < 0.05).

## 4. Discussion

In the present study, there were five notable findings for long COVID patients with headaches. For long COVID patients with headaches, (1) the median age of the patients was younger than that of patients without headaches, (2) the time lag before the first visit to a long COVID clinic was shorter than that for patients without headaches, and (3) the proportion of patients in the Omicron-variant phase was larger than the proportions of patients in other phases. Among the various symptoms of long COVID, (4) general fatigue, insomnia, dysosmia, dizziness, fever, and chest pain were frequent in the patients with headaches. Finally, (5) the manifestation of headache was linked to deterioration of social and psychological activities assessed by scores for depression, QOL, and general fatigue.

Among the 482 patients with long COVID who visited our outpatient clinic, 113 (23.4%) complained of headaches. It was shown in a meta-analysis of 35 studies for 28,348 survivors that the prevalence of long COVID-related headaches was higher in patients that were managed in an outpatient setting during the acute phase [25]. In those studies, 14% to 60% of the patients had headaches during the acute COVID-19 phase [26,27], whereas the prevalence of persistent headaches in long COVID patients was 18%, being more prevalent in middle-aged women, and the headaches started two weeks after respiratory symptoms had subsided [11,13]. In our study on long COVID patients with headaches, female predominancy (F/M: 56%/44%) and younger age (F/M: 35/35 years) with relatively early visits after the onset of infection (F/M: 83/87 days) were revealed compared with the headache-free patients (F/M: 41/41 years; duration before visits: F/M: 114/112 days).

Our present study also revealed that patients with headaches were more likely to have other complications, including general fatigue, insomnia, dysosmia, dizziness, fever, and chest pain. Headache is one of the most disabling symptoms of long COVID. It may occur alone or in combination with other symptoms of long COVID, such as muscle weakness, dizziness, and vertigo, as well as insomnia or other sleep impairments [14,15]. It has also been shown that approximately 14% of the patients examined had long-COVID headaches in a case-control analysis nested in a prospective cohort study [9], in which the patients with long-COVID headaches were younger and female predominant and had higher frequencies of anosmia and myalgia. Another cohort study further showed that a large number of patients with primary headaches had a worsening of their headaches within 3 months after the onset of COVID-19 [28]. The reason for the earlier hospital visits by long COVID patients with headaches might be not only the direct effect of persistent headaches but also the effects of other co-existing symptoms.

As for the various conditions related to headaches, it is important to diagnose headaches in the following order: urgent secondary headache, possible secondary headache, common primary headache, and rare primary headache [29]. The possibility of a secondary headache should always be considered before diagnosing a primary headache. In cases of headaches associated with COVID-19, headaches accompanying neurological findings and symptoms, fever, and weight loss should be carefully examined [30,31]. Based on the results of the present study, headaches with comorbid symptoms, including general fatigue, insomnia, dizziness, fever, and chest pain, and also with depressive status, are likely to be characteristic for the diagnosis of secondary headache due to long COVID.

During the COVID-19 pandemic, it was shown that the incidence of headaches increased up to five times, and the pathogenesis underlying persistent headaches might be attributed to a cytokine storm with persistent activation of the immune system as indicated by changes in the circulating levels of various cytokines and interleukins [32]. Although critical biomarkers for headaches related to long COVID were not identified in our study, very slight increases in serum thyroid hormone and IGF-I levels might indicate the presence of a thyrotoxic condition related to headache stress. On the other hand, cardiopulmonary dysfunction does not seem to be a causal factor for long-COVID headaches [9]. Clinically, a headache may manifest as a migraine or a tension-type phenotype [11]. Namely, de novo headache is a common post-COVID-19 symptom and can persist long after the resolution of the infection. Post-COVID-19 headaches often have migraine features with the concomitant complication of anosmia [28,33], in which persistent glial activation and trigeminal-vascular stimulation have been considered to play a role [33,34] by inflammation or direct involvement of SARS-CoV-2.

In the present study, we found that the QOL of COVID-19 survivors is greatly influenced by the existence of headaches since the manifestation of headaches has been linked to declines in social and psychological activities. Given that the pandemic condition itself could induce increased levels of emotional and physical stress, it is possible that the pandemic stress can be a risk for the progression and development of various types of headaches. Further clinical and basic approaches are necessary to clarify the fundamental characteristics of long COVID headaches.

The current study has several limitations. Firstly, this study was a Japanese single-center retrospective study. Since the patients were consulted in hospitals in various prefectures in Japan, the cases with relatively severe symptoms tended to be referred. Therefore, multicenter prospective studies are needed to prove a causal relationship between headaches and long COVID symptoms. Secondly, patient comorbidities and background factors were not considered in our study. Thirdly, since headaches can be described in various ways, they may include multiple symptoms, such as brain fog symptoms. Fourthly, due to the study design, it was difficult to differentiate the clinical types of various headaches in this study. Further research and data consideration is needed to determine the biological mechanism of the increased frequency of headaches in long COVID patients.

In conclusion, it was shown in the present study that about one-fourth of the long COVID patients had headaches. The long COVID patients with headaches were younger, tended to have Omicron-dominant infection, and visited the clinic earlier. Symptoms, including general fatigue, insomnia, dizziness, fever, and chest pain, tended to be enhanced by headaches, leading to the deterioration of QOL of the long COVID patients. Since the manifestation of headaches was shown to have a significant impact on social and psychological activities, we need to pay attention to headaches as a priority for treating long COVID.

## Figures and Tables

**Table 1:** Backgrounds of long COVID patients with headaches.

Patient Groups	Headache-Free Group (*n* = 369, 76.6%)	Headache Group (*n* = 113, 23.4%)	*p* Value
Age (years), median (IQR)	42 (28–52)	37 (22–45)	<0.01 **
Height (cm), median (IQR)	163.0 (155.8–170.4)	163.7 (156.7–170.3)	0.629
Weight (kg), median (IQR)	61.3 (52.5–72.2)	61.8 (52.8–73.0)	0.771
BMI, median (IQR)	22.6 (20.4–48.7)	23.2 (20.5–26.5)	0.619
Gender			
Female, *n* (%)	199 (53.9%)	63 (55.8%)	0.816
Male, *n* (%)	170 (46.1%)	50 (44.2%)	
Smoking habit, *n* (%)	125 (33.9%)	42 (37.2%)	0.596
Alcohol drinking habit, *n* (%)	158 (42.8%)	45 (39.8%)	0.649
Severity of COVID-19			
Mild, *n* (%)	305 (82.7%)	100 (88.5%)	0.199
Moderate, *n* (%)	58 (15.7%)	13 (11.5%)	
Severe, *n* (%)	6 (1.6%)	0 (0.0%)	

Medians [IQR: interquartile ranges] and percentages (%) are shown. BMI: body mass index, COVID-19: coronavirus disease 2019. The Mann-Whitney U test and χ^2^ test were performed when appropriate for statistics, and ** *p* < 0.01 indicates statistically significant differences.

## Data Availability

Detailed data available if requested from the corresponding author.

## Abbreviations

coronavirus disease 2019 (COVID-19); COVID-19 aftercare clinic (CAC); Euro QOL 5-dimensions 5-levels (EQ-5D-5L); fatigue assessment scale (FAS); frequency scale for the symptoms of gastroesophageal reflux disease (FSSG); post COVID-19 condition (PCC); quality of life (QOL); self-rating depression scale (SDS); severe acute respiratory syndrome coronavirus 2 (SARS-CoV-2).
